# Arrhythmic events associated with immune checkpoint inhibitors therapy: A real‐world study based on the Food and Drug Administration Adverse Event Reporting System database

**DOI:** 10.1002/cam4.5438

**Published:** 2022-11-24

**Authors:** Yunwei Liu, Yanxin Chen, Zhimin Zeng, Anwen Liu

**Affiliations:** ^1^ Department of Oncology The Second Affiliated Hospital of Nanchang University Nanchang Jiangxi China; ^2^ Jiangxi Key Laboratory of Clinical Translational Cancer Research The Second Affiliated Hospital of Nanchang University Nanchang Jiangxi China; ^3^ Radiation Induced Heart Damage Institute of Nanchang University Nanchang Jiangxi China

**Keywords:** adverse drug events, arrhythmic events, data mining, FAERS, immune checkpoint inhibitors

## Abstract

**Background:**

Although arrhythmias have been reported in patients treated with immune checkpoint inhibitors (ICIs), the association between arrhythmias and ICIs has not been thoroughly evaluated in real‐world studies. We aimed to describe the major features of ICI‐related arrhythmic events and identify the factors that contributed to death.

**Methods:**

A disproportionality analysis was performed using data from the Food and Drug Administration Adverse Event Reporting System (FAERS) database from January 2011 to December 2021. Reporting odds ratios (RORs), proportional reporting ratio and information component were used to assess whether adverse arrhythmic events were associated with ICIs. The clinical characteristics of patients with ICI‐associated arrhythmias were compared with fatal and non‐fatal arrhythmias. The time to onset (TTO), fatality rates of arrhythmic events were also investigated.

**Results:**

We identified a total of 1945 cases of ICI‐related arrhythmic events. Men (64.78%) were identified significantly more frequently than women (28.84%). The median age was 68 years ([interquartile range, IQR] 60–75 years). Anti‐programmed cell death‐1 (PD‐1) and anti‐programmed cell death ligand‐1 (PD‐L1) were associated with adverse arrhythmic events, corresponding to ROR 1.11 (95% confidence interval [CI] 1.05–1.17) and ROR 1.34 (95% CI 1.20–1.49), respectively. However, anti‐cytotoxic T‐lymphocyte associated protein 4 or combination immunotherapy did not appear to be associated with arrhythmic events. Atrial fibrillation (*N*  = 576, 0.62%), cardiac arrest (*N*  = 284, 0.31%), tachycardia (*N*  = 175, 0.19%) were the most common adverse arrhythmic events. Sudden death and complete atrioventricular block are adverse events that are significantly associated with ICI‐related arrhythmic events and have strong signal intensity. The TTO of cases that resulted in death (30 days [IQR] 11–73.75) was significantly earlier than that of cases that did not result in death (33 days [IQR 10.5–88.5], *p*  = 0.003). ICI‐related arrhythmic events were severe with death occurring in 507 (26.07%) of 1945 arrhythmias cases.

**Conclusions:**

Treatment with PD‐1/PD‐L1 may cause arrhythmic events, which are severe and tend to occur early on during treatment. It is important to identify ICI‐related arrhythmias as early as possible, and to manage them appropriately.

## Graphical abstract

This is the largest and most comprehensive pharmacovigilance study until recently to compare the clinical features, associations, timing and prognosis of ICIs‐related arrhythmic events in the real‐world study based on the Food and Drug Administration Adverse Event Reporting System (FAERS) database. Our study shows anti‐PD‐1/PD‐L1 monotherapy may cause arrhythmic events, which are severe and tend to occur early on during treatment. It is important to identify ICI‐related arrhythmias as early as possible, and to manage them appropriately.

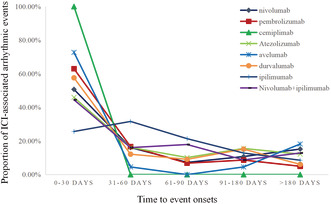

## 1 INTRODUCTION

In recent years, due to an in‐depth understanding of the tumor immune microenvironment and the mechanism of tumor immune escape, treatment by targeted inhibition of immune checkpoint molecules such as programmed cell death‐1 (PD‐1)/programmed cell death ligand‐1 (PD‐L1) and cytotoxic T‐lymphocyte associated protein 4 (CTLA‐4) has brought a revolutionary change to the treatment of malignant tumors.^1^, ^2^ Immune checkpoint inhibitors (ICIs) significantly improve the prognosis of patients in the treatment of malignant tumors by activating the killing effect of CD8^+^ T lymphocytes.^3^ Therefore, ICIs have been gradually developed and are widely used on a variety of malignant tumors.^4^ Currently, the US Food and Drug Administration (FDA) has approved seven ICIs, including monoclonal antibodies targeting PD‐1 (nivolumab, pembrolizumab, cemiplimab), PD‐L1 monoclonal antibodies (atezolizumab, avelumab, durvalumab), and CTLA‐4 (ipilimumab, tremelimumab).^5^

By blocking immune checkpoint molecules, ICIs reverse the inhibition of T lymphocyte function by tumors and restore the killing effect of the immune system on tumor cells.^6^ However, while activated T cells have antitumor effects, they may also attack the normal tissues of the body and induce immune‐related adverse events (irAEs).^7^ Among them, the incidence of ICI‐associated myocarditis is low, but its fatality rate is as high as 50%.^8^, ^9^, ^10^ ICI‐associated myocarditis has become an unavoidable challenge in clinical practice due to its extremely high fatality rate, and arrhythmia is a clinical manifestation of ICI‐associated myocarditis. Previous studies have shown that various types of arrhythmias have been reported in patients treated with ICIs; the most common is atrial fibrillation, followed by ventricular tachycardia or ventricular fibrillation, and atrioventricular (AV) block, among which complete AV block and ventricular tachyarrhythmias are fatal.^11^ Due to an insufficient understanding of the forms of arrhythmias associated with ICIs, data on arrhythmias are mainly derived from case reports,^12^, ^13^, ^14^ small retrospective studies, and clinical trials. Due to the inherent limitations of case reports, retrospective studies, and clinical trials (including limited sample size, lack of follow‐up), real‐world conditions may not be accurately represented by these sources. In addition, the characteristics, timing, outcomes, and death‐related factors associated with ICI‐related arrhythmic events remain unclear.

There is a pressing need to identify the clinical features of ICIs‐associated arrhythmic events, since they are widely used for treating tumors and causing potentially fatal arrhythmias. With the expansion of the indications for the use of ICIs and the increasing use of their combination with other treatments, a systematic study of arrhythmic events associated with ICIs can help clinicians fully understand the adverse arrhythmic events induced by ICIs, and it is of great significance to warn of their drug safety. Therefore, we performed a pharmacovigilance analysis based on the US Food and Drug Administration Adverse Event Reporting System (FAERS) database to describe and assess adverse arrhythmic events induced by different ICI regimens. In addition, we further explored death‐related factors and their main characteristics to provide a reference for their safe use in clinical practice.

## 2 METHODS

### 2.1 Data source

Based on the FAERS database, we conducted a retrospective pharmacovigilance analysis. The adverse drug event reports in the FAERS database come from voluntary reports by doctors, pharmacists, patients and pharmaceutical companies around the world. This database is the world's main spontaneous reporting system of adverse drug events, and it is a free and publicly accessible database. FAERS data include basic patient information, adverse reaction information, drug use, reporting source information, the start and end times of drug treatment, drug indications, and patient treatment outcomes. This study screened 44 quarterly documents of the FAERS database (https://fis.fda.gov/extensions/FPD‐QDE‐FAERS/FPD‐QDE‐FAERS.html) from Q1 2011 to Q4 2021.

### 2.2 Procedures

Due to FAERS's lack of a uniform coding system for drug names, we searched for ICIs using FDA‐approved brand names and generic names (Table S1), and selected “PS” (primary suspect) under the role_cod field. Medical Dictionary for Regulatory Activities (MedDRA) Preferred Terms are used to code FAERS reports. After reviewing the literature, summarizing previous studies, and standardizing MedDRA queries, we concluded that the following preferred terms (PTs) were associated with arrhythmias: arrhythmia, tachycardia, bradycardia, tachyarrhythmia, sinus tachycardia, sinus bradycardia, atrial flutter, atrial fibrillation, arrhythmia supraventricular, supraventricular tachycardia, ventricular arrhythmia, ventricular tachycardia, ventricular extrasystoles, ventricular fibrillation, conduction disorder, AV block, AV block second degree, AV block complete, bundle branch block left, bundle branch block right, electrocardiogram qt prolonged, sudden death, cardiac arrest, and sudden cardiac death. The clinical characteristics of patients with ICI‐related arrhythmic events (sex, age, reporting time, reporting area, primary tumor site, outcome, time of starting medication, time of adverse events [ADEs], etc.) A comparison was made between patients with fatal and non‐fatal ICI‐related arrhythmic events based on their clinical characteristics. Moreover, we evaluated the time to onset (TTO) of arrhythmic events caused by different ICIs. The time interval between START_DT (ICI use start date) and EVENT_DT (adverse event occurrence date) was defined as TTO, and incorrect records were excluded. In addition, we also counted the reports of fatal events caused by ICIs. The proportion of deaths was the number of fatal results divided by the total number of reported events.

### 2.3 Statistical analysis

Clinical characteristics of the cases were summarized using descriptive statistics. Chi‐square tests were used to compare categorical variables between groups and *t* tests and nonparametric tests were used for continuous variables with normal and nonnormal distributions, respectively. *p* < 0.05 was deemed statistically significant.

In this study, we mainly used the reporting odds ratio (ROR), proportional reporting ratio (PRR) and Bayesian confidence propagation neural networks of information components (IC) to mine adverse reaction signals. Previous studies have described in detail data mining theories and formulas,^15^, ^16^ and the calculation formulas are shown in Tables S2 and S3. Using the above algorithm, we compared the correlation between the various ICIs and arrhythmic events. If an IC025 (the lower limit of the IC 95% confidence interval [CI]) value was greater than zero or the lower end of the 95% CI of ROR was greater than one and the number of reported cases was ≥3 or PRR was greater than one and *χ* ^2^ (chi‐square) was greater than four and the number of reported cases was ≥3, then we considered that a potential ICI‐related adverse arrhythmic reaction signal had been generated.

A significant signal indicates a relationship between a given drug and adverse effects, but it cannot be regarded as causality or a reliable surrogate for morbidity. The data analysis was performed using R (Version 4.0.5), OpenVigil 2.1 and SPSS 24.0 (SPSS, Inc.). OpenVigil is open tools for data‐mining and analysis of pharmacovigilance data.^17^

## 3 RESULTS

### 3.1 Descriptive analysis

From January 2011 to December 2021, a total of 227,033 ICI‐related ADEs and 308,385 arrhythmia‐related reports were recorded in the FAERS database. As shown in Table 1, we screened 1945 reports of suspected ICI‐related arrhythmic events and summarized the clinical characteristics of these patients. The majority of reported cases (64.78%) were males. More than half of the reported cases were aged ≥65 years, with a median age of 68 years. Among the countries with the most cases, the American continent accounted for 42.88%, followed by Europe (33.11%) and Asia (20.93%). Adverse arrhythmic events were most common in lung cancer patients (32.08%), followed by melanoma (20.46%) and urinary system tumors (16.25%). Comparison of clinical characteristics of ICI‐related arrhythmia events between lung cancer patients and patients with other indications is detailed in Table S4. Hospitalization (48.02%) was the most common outcome, with death accounting for 26.07%. The median time of adverse arrhythmic events was 32 days, and 48.90% of ADEs occurred within 30 days. The most reported arrhythmia was in response to nivolumab monotherapy (39.13%), followed by pembrolizumab monotherapy (19.28%).

**TABLE 1:** Clinical characteristics of patients with arrhythmic events associated with immune checkpoint inhibitors

Characteristics	Total reports, *n* (%)	Fatal cases, *n* (%)	Non‐fatal cases, *n* (%)	*p*‐value
Total	1945	507	1438	
Gender				NS
Female	561 (28.84)	137 (27.02)	424 (29.49)	
Male	1260 (64.78)	337 (66.47)	923 (64.19)	
Unknown	124 (6.38)	33 (6.51)	91 (6.33)	
Age				<0.0001
Median	68 (60–75)	68 (59.25–75)	68 (61–75)	
<18	7 (0.36)	0	7 (0.49)	
18–64	554 (28.48)	147 (28.99)	407 (28.30)	
≥65	1050 (53.98)	269 (53.06)	781 (54.31)	
Unknown	334 (17.17)	91 (17.95)	243 (16.90)	
Reporting year				NS
2011	19 (0.98)	7 (1.38)	12 (0.83)	
2012	30 (1.54)	8 (1.58)	22 (1.53)	
2013	24 (1.23)	5 (0.99)	19 (1.32)	
2014	36 (1.85)	8 (1.58)	28 (1.95)	
2015	81 (4.16)	23 (4.54)	58 (4.03)	
2016	129 (6.63)	43 (8.48)	86 (5.98)	
2017	234 (12.03)	55 (10.85)	179 (12.45)	
2018	314 (16.14)	86 (16.96)	228 (15.86)	
2019	345 (17.74)	85 (16.77)	260 (18.08)	
2020	323 (16.61)	80 (15.98)	243 (16.90)	
2021	410 (21.08)	107 (21.10)	303 (21.07)	
Reporting region				<0.0001
Europe	644 (33.11)	154 (30.37)	490 (34.08)	
America	834 (42.88)	186 (36.69)	648 (45.06)	
Asia	407 (20.93)	160 (31.56)	247 (17.18)	
Oceania	53 (2.72)	7 (1.38)	46 (3.20)	
Africa	4 (0.21)	0	4 (0.28)	
Unknown	3 (0.15)	0	3 (0.21)	
Indications (Top 10)				<0.01
Lung cancer	624 (32.08)	175 (34.52)	452 (31.43)	
Melanoma	398 (20.46)	92 (18.15)	306 (21.28)	
Tumors of urinary system	316 (16.25)	75 (14.79)	241 (16.76)	
Head and neck cancer	71 (3.65)	32 (6.31)	39 (2.71)	
Hematological cancer and lymphoma	56 (2.88)	12 (2.37)	44 (3.06)	
Tumors of female reproductive organs	47 (2.42)	16 (3.16)	31 (2.16)	
Gastrointestinal cancer	44 (2.26)	17 (3.35)	27 (1.88)	
Breast cancer	32 (1.65)	6 (1.18)	26 (1.81)	
Mesothelioma	28 (1.44)	2 (0.39)	26 (1.81)	
Hepatocellular carcinoma	22 (1.13)	9 (1.78)	13 (0.90)	
Outcome				NS
Death	507 (26.07)	507 (100.00)	0	
Life‐threatening	150 (7.71)		150 (10.43)	
Disability	25 (1.29)		25 (1.74)	
Hospitalization	934 (48.02)		934 (64.95)	
Other outcomes	299 (15.37)		299 (20.79)	
RI	6 (0.31)		6 (0.42)	
CA	1 (0.05)		1 (0.07)	
Non‐serious	23 (1.18)		23 (1.60)	
Reporter TTO				0.003
Median	32 (11–84)	30 (11–73.75)	33 (10.5–88.5)	
0–30	558 (28.69)	154 (30.37)	404 (28.09)	
31–60	199 (10.23)	54 (10.65)	145 (10.08)	
61–90	125 (6.43)	37 (7.30)	88 (6.12)	
91–180	124 (6.38)	26 (5.13)	98 (6.82)	
>180	135 (6.94)	29 (5.72)	106 (7.37)	
Unknown	804 (41.34)	207 (40.83)	597 (41.52)	
ICI drug as suspected drug				NS
Monotherapy	1631(83.86)	430 (84.81)	1201 (83.52)	NS
Anti‐PD‐1 monotherapy	1144 (58.82)	304 (59.96)	840 (58.41)	
Pembrolizumab	375 (19.28)	105 (20.71)	270 (18.78)	
Nivolumab	761 (39.13)	197 (38.86)	564 (39.22)	
Cemiplimab	8 (0.41)	2 (0.39)	6 (0.42)	
Anti‐PD‐L1 monotherapy	309 (15.89)	80 (15.78)	229 (15.92)	
Atezolizumab	224 (11.52)	54 (10.65)	170 (11.82)	
Avelumab	29 (1.49)	7 (1.38)	22 (1.53)	
Durvalumab	56 (2.88)	19 (3.75)	37 (2.57)	
Anti‐CTLA‐4 monotherapy	178 (9.15)	46 (9.07)	132 (9.18)	
Ipilimumab	178 (9.15)	46 (9.07)	132 (9.18)	
Tremelimumab	0	0 (0.00)	0	
Combination therapy	314 (16.14)	77 (15.19)	237 (16.48)	NS
Ipilimumab + nivolumab	288 (14.81)	69 (13.61)	219 (15.23)	
Ipilimumab + pembrolizumab	5 (0.25)	1 (0.20)	4 (0.28)	
Tremelimumab + durvalumab	9 (0.46)	4 (0.79)	5 (0.35)	
Pembrolizumab + atezolizumab	12 (0.62)	3 (0.59)	9 (0.63)	

Abbreviations: CA, congenital anomaly; CTLA, cytotoxic T‐lymphocyte associated protein 4; ICI, immune checkpoint inhibitor; PD‐1, programmed cell death‐1; PD‐L1, programmed cell death ligand‐1; RI, required intervention; TTO, time to onset.

### 3.2 Immune therapy‐related signal values

In Table 2, we show the signals and the association between total/class‐specific ICI and arrhythmic events. Overall, the reported frequency of adverse arrhythmic events were significantly associated with ICI treatment (ROR025 = 1.08, IC025 = 0.11). In terms of the different classes of ICIs, there was also a significant association between arrhythmia and anti‐PD‐1 drugs and anti‐PD‐L1 drugs. Most arrhythmic ADEs occurred in patients treated with anti‐PD‐1 drugs (58.82%), especially nivolumab (39.13%). Despite being reported less frequently (15.89%), anti‐PD‐L1 drugs showed stronger signals (ROR025 = 1.20, IC025 = 0.27). Signal values for avelumab were the strongest (ROR025 = 1.43, IC025 = 0.51). However, anti‐CTLA‐4 drugs were not significantly associated with adverse cardiac arrhythmia events (ROR025 = 0.90, IC025 = −0.15). Ipilimumab plus nivolumab was the most common combination therapy (14.81%), and it is worth noting that the combination of ICIs was not associated with adverse arrhythmic events. In our subsequent analysis, however, anti‐CTLA‐4 drugs as well as ipilimumab plus nivolumab were significantly associated with a number of specific adverse arrhythmic events.

**TABLE 2:** Signal detection for ICIs‐associated arrhythmic events

Strategy	Drug	*N*	ROR (95% CI)	PRR (*χ* ^2^)	IC (95% CI)
Total	Total ICIs	2142	1.12 (1.08, 1.17)	1.12 (28.44)	0.17 (0.11, 0.23)
Monotherapy	Anti‐PD‐1	1275	1.11 (1.05, 1.17)	1.10 (12.50)	0.14 (0.06, 0.22)
	Pembrolizumab	423	0.93 (0.85, 1.02)	0.93 (2.14)	−0.10 (−0.24, 0.03)
	Nivolumab	844	1.23 (1.15, 1.32)	1.23 (35.32)	0.30 (0.20, 0.40)
	Cemiplimab	8	0.66 (0.33, 1.32)	0.66 (1.09)	−0.60 (−1.61, 0.40)
	Anti‐PD‐L1	341	1.34 (1.20, 1.49)	1.33 (28.42)	0.42 (0.27, 0.57)
	Atezolizumab	250	1.45 (1.28, 1.64)	1.45 (34.13)	0.54 (0.36, 0.72)
	Avelumab	32	2.02 (1.43, 2.87)	2.01 (15.26)	1.02 (0.51, 1.52)
	Durvalumab	59	0.88 (0.68, 1.14)	0.88 (0.78)	−0.18 (−0.55, 0.19)
	Anti‐CTLA‐4	186	1.04 (0.90, 1.21)	1.04 (0.30)	0.06 (−0.15, 0.27)
	Ipilimumab	186	1.04 (0.90, 1.21)	1.04 (0.30)	0.06 (−0.15, 0.27)
	Tremelimumab	0			
Anti‐PD‐1 vs. anti‐PD‐L1			0.83 (0.73, 0.93)		
Anti‐PD‐1 vs. anti‐CTLA‐4			1.06 (0.91, 1.24)		
Anti‐PD‐L1 vs. anti‐CTLA‐4			1.28 (1.07, 1.53)		
Combination therapy		340	1.06 (0.95, 1.18)	1.06 (1.11)	0.09 (−0.07, 0.24)
	Ipilimumab + nivolumab	307	1.05 (0.94, 1.17)	1.05 (0.63)	0.07 (−0.09, 0.23)
	Ipilimumab + pembrolizumab	6	0.58 (0.26, 1.29)	0.58 (1.42)	−0.79 (−1.94, 0.37)
	Tremelimumab + durvalumab	9	1.85 (0.96, 3.58)	1.84 (2.67)	0.89 (−0.06, 1.84)
	Pembrolizumab + atezolizumab	18	1.44 (0.90, 2.29)	1.43 (1.97)	0.53 (−0.15, 1.19)
Combination vs. monotherapy therapy			0.93 (0.83, 1.05)		

Abbreviations: *χ* ^2^, chi‐squared; CI, confidence interval; CTLA, cytotoxic T‐lymphocyte associated protein 4; IC, information components; *N*, number of records; PD‐1, programmed cell death‐1; PD‐L1, programmed cell death ligand‐1; PRR, proportional reporting ratio; ROR, reporting odds ratio.

### 3.3 The signal spectrum of arrhythmia differs in immune therapies

**FIGURE 1:**
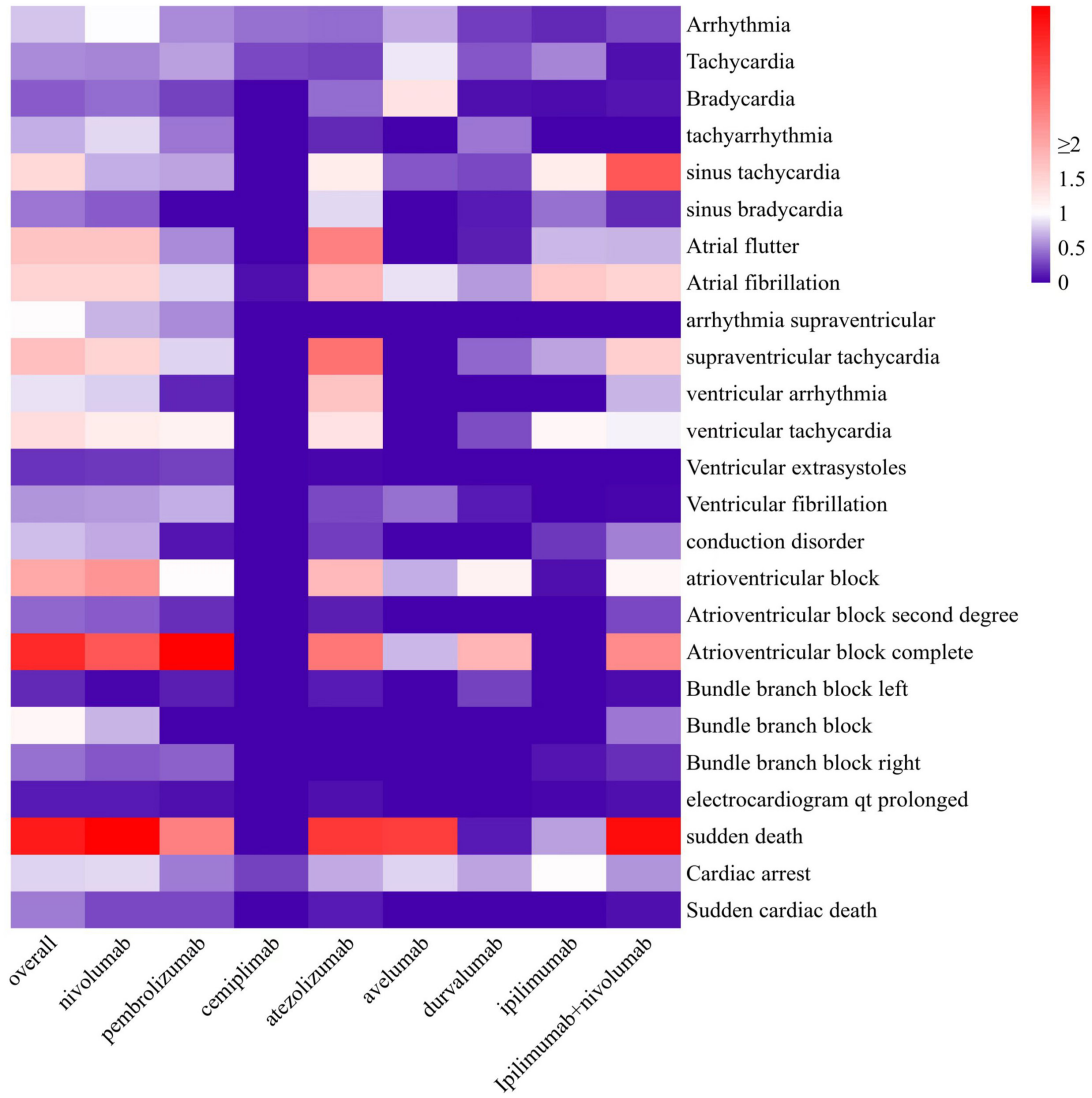
Arrhythmia signal profiles of different ICI strategies. The x axis represents different ICI strategies, y axis represents arrhythmic events; ROR025: the lower end of the 95% confidence interval of ROR. Statistically significant RORs (i.e., ROR025 >1) are assigned with a red color. ICI, immune checkpoint inhibitor; ROR, reporting odds ratio

Due to the low number of reported cases of ICI‐related arrhythmias, if data mining is limited to a general level, important signals may be overlooked. Therefore, we investigated whether each specific adverse arrhythmic event is associated with specific ICIs. The lower end of the 95% CI of ROR (ROR025) is used as an indicator in Figure 1 to show the arrhythmia signal spectrum for various ICI strategies. Overall, 10 PTs were significantly associated with ICIs, 7 PTs with nivolumab, 4 PTs with pembrolizumab, and 6 PTs with ipilimumab plus nivolumab. In contrast, only 2 PTs were associated with avelumab and durvalumab, and no PTs were associated with cemiplimab. Cemiplimab is only used for the treatment of metastatic cutaneous squamous cell carcinoma (CSCC) or locally advanced CSCC that cannot be treated with curative surgery or radiotherapy,^18^ and its rare use results in only a small number of reported ADEs. Therefore, cemiplimab is rarely analyzed and discussed in other studies. Table S5 shows atrial fibrillation (*N* = 576, 0.62%), Cardiac arrest (*N* = 284, 0.31%), Tachycardia (*N* = 175, 0.19%) were the top three adverse arrhythmic events reported in the database. Notably, sudden death and complete AV block were the ADEs that were significantly associated with ICI‐related arrhythmic events, and they had stronger signal intensity.

### 3.4 Analysis of fatal and non‐fatal cases

Table 1 shows that significant difference in fatal and non‐fatal cases was not observed based on sex, reporting year, etc. Fatal arrhythmic events are more likely to occur in patients aged 65 years or older. There are also differences in the mortality rate associated with different primary lesions; fatal arrhythmic events were more common in lung cancer and malignant melanoma, accounting for 28.04% and 23.10%, respectively. In addition, a significant difference (*p* < 0.001) was found between fatal and non‐fatal cases across reporting regions, with Asia reporting the highest proportion of deaths (39.31%, 160/407). In cases of fatal and non‐fatal ICI‐associated arrhythmic events, the proportion of fatal arrhythmias and non‐fatal arrhythmias was similar between ICI monotherapy and ICI combined therapy. Notably, there was a significant difference in TTO between the two groups, with fatal cases having a shorter TTO than non‐fatal cases (median 30 days [IQR 11.00–73.75 days] and 33 days [IQR 10.5–88.5 days], respectively; *p* = 0.003).

### 3.5 TTO of ICI‐related arrhythmic events

**FIGURE 2:**
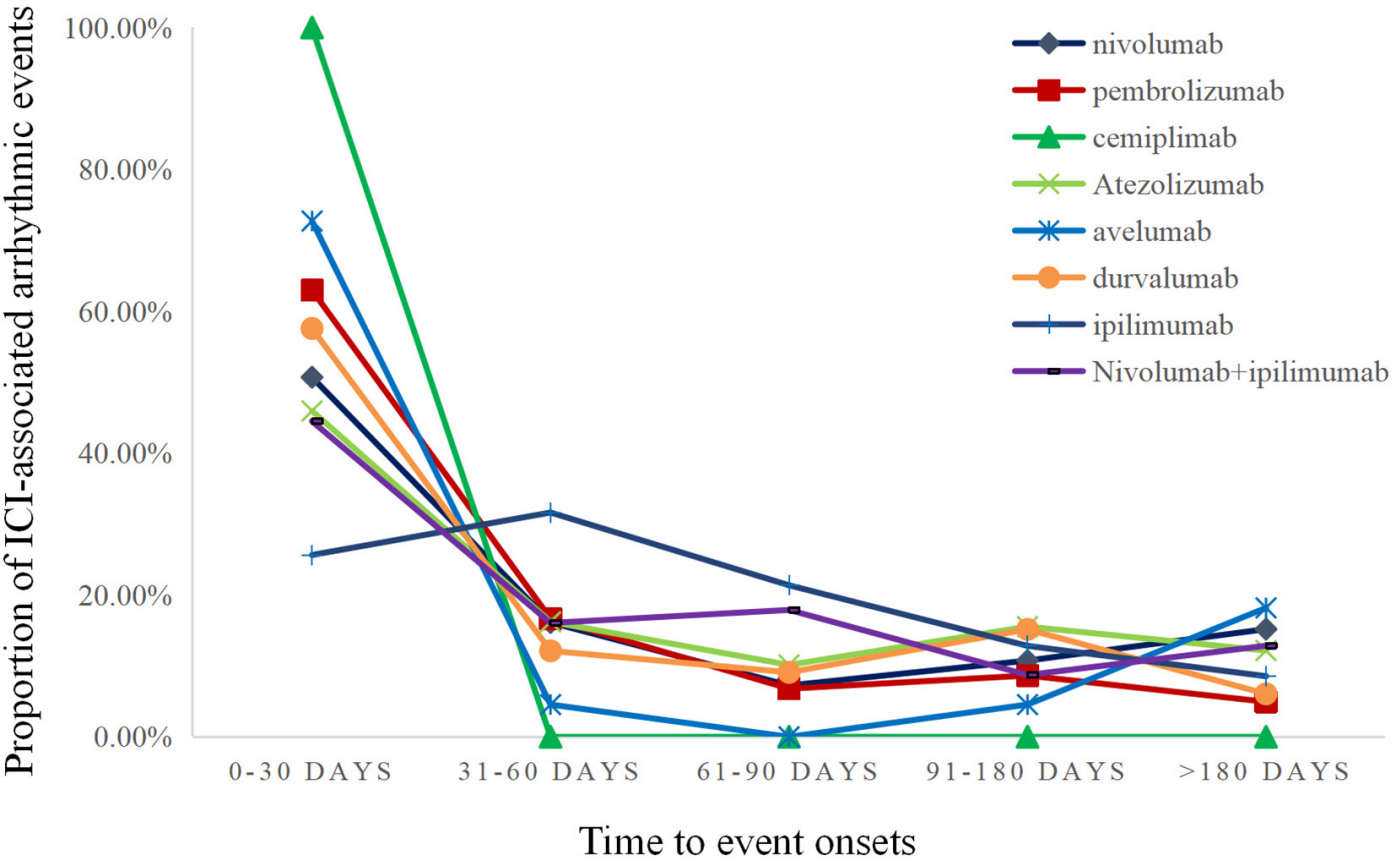
Time to event onset of arrhythmic events following different immune checkpoint inhibitor regimens.

The median TTO for ICI‐related arrhythmic events was 32 days (IQR 11–84 days). Figure 2 shows the onset time of ICI‐related arrhythmic events under each ICI regimen (ipilimumab plus pembrolizumab, tremelimumab plus durvalumab, and pembrolizumab plus atezolizumab have few data points and are not shown in Figure 2). A significant difference was found between ICI regimens at the time of arrhythmia onset (*p* < 0.0001). As for the median TTO of arrhythmic events, avelumab exhibited the shortest median time 1 (IQR 0–63) days, while ipilimumab exhibited the longest 47 (IQR 29.5–83.5) days. Furthermore, the median TTO was 29 (IQR 8.00–97.75) days for nivolumab, 20 (IQR 2.00–51.75) days for pembrolizumab, 20.5 (IQR 18.50–26.25) days for cemiplimab, 39.5 (IQR 13–102) days for atezolizumab, 29 (IQR 7–90) days for durvalumab and 37.5 (IQR 16–84) days for ipilimumab plus nivolumab. According to the data, patients who received ipilimumab plus nivolumab had an earlier TTO (37.5 [IQR 16–84]) than those who received ipilimumab alone(49 [IQR 29.5–83.5]) (*p* < 0.001).

### 3.6 Fatality rates

**FIGURE 3:**
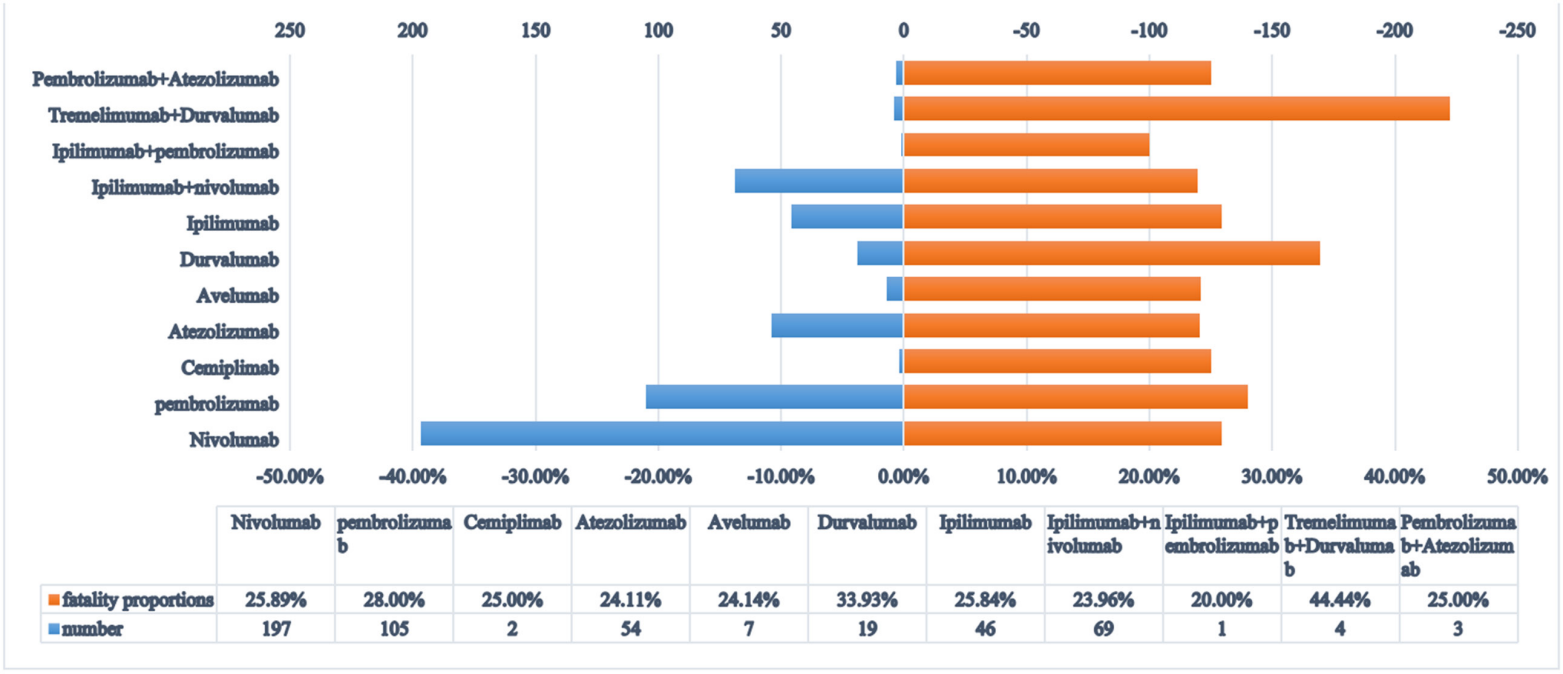
Number of reports and fatality rates for immune checkpoint inhibitor‐associated arrhythmic events. Number: the number of reports of death. Fatality proportions: the proportion of death outcomes in all reported outcomes.

To determine the prognosis of ICI‐treated patients who have adverse arrhythmic events, we assessed the rate of arrhythmia‐related deaths, as shown in Figure 3. According to our analysis, the fatality rate of ICI‐related arrhythmic events was 26.07%. Among all monotherapies, durvalumab had the highest fatality rate (33.93%), followed by pembrolizumab (28.00%), and atezolizumab had the lowest fatality rate (24.11%). In combination therapy, the death rate of tremelimumab + durvalumab was as high as 44.44%, but due to the small number of reports, it is not enough to draw conclusions. Moreover, we further classified these arrhythmia events according to adverse drug reactions encoded in the MedDRA.^15^ The fatality rates of common ICI‐related arrhythmia was analyzed, as Table 3 shows, cardiac arrest (*N* = 299, 66.89%), conduction disease (*N* = 47, 23.50%), Ventricular arrhythmia (*N* = 43, 14.34%) were the top three adverse arrhythmic events reported in the database. The comparison of clinical features between cardiac arrest and other arrhythmia event reports in ICI‐related arrhythmia event reports is detailed in Table S6. All other combination treatments had lower mortality rates than monotherapy.

**TABLE 3:** The fatality rates of common ICI‐related arrhythmic events

Arrhythmic event	*N*	%
Cardiac arrest	299	66.89
Conduction disease	47	23.50
Ventricular arrhythmia	43	20.98
Supraventricular tachycardia	104	14.34
QT prolongation	2	8.33

Abbreviation: *N*, number of death records.

## 4 DISCUSSION

Myocarditis inevitably occurs in tumor patients treated with ICIs, which affects the efficacy of immunotherapy and even threatens their lives; thus, it deserves further attention. In addition to life‐threatening myocarditis, these fatal cardiovascular toxicities include cardiac arrhythmias and pericardial disease. Currently, most studies focus only on specific adverse cardiac events, such as myocarditis, pericardial disease, vasculitis, or broad‐spectrum cardiotoxicity.^19^, ^20^, ^21^, ^22^, ^23^, ^24^ However, there are few studies on ICI‐related arrhythmias, and the relationship between different ICIs and arrhythmic events is rarely reported. To identify and assess the relationship between ICIs and arrhythmic events in the real world, we collected data from the FAERS database on ICI‐associated arrhythmic events, which can provide a reference for future prevention and treatment. We screened 1945 cases of arrhythmic events associated with ICIs, the largest collection of such cases to date. Arrhythmias following treatment with ICIs have increased annually over time, reflecting the widespread use of ICIs in oncology treatment and increased awareness among health care professionals of post‐market surveillance of ICIs. Our findings are as follows:

The sex distribution of patients with ICI‐related arrhythmic events showed that male patients were more likely to develop arrhythmias than female patients, which was consistent with a previous study on ICI‐related cardiotoxicity.^23^ Possible causes include several factors: First, the real world does not recommend ICIs for women, and they are often excluded from clinical trials.^25^, ^26^ Second, for all ICIs, the main therapeutic indications are melanoma, lung cancer, renal cell carcinoma, bladder cancer, head and neck cancer, etc., and the fact that the incidence of these malignancies is higher in males than in females leads to treatment with ICIs in a clinical setting for a larger proportion of men than of women.^27^, ^28^ However, Jing et al.^29^ found that there were no differences in irAE risk, related factors or pathways between cancer patients of different sexes treated with ICIs. Therefore, it is necessary to conduct further studies to determine the true incidence rate of ICI‐related arrhythmias among men and women. Based on the results of this study, ICI‐associated arrhythmias were more common in elderly patients (53.98% ≥65 years vs. 28.84% <65 years) with a median age of 68 years. Age is one of the important risk factors for cancer, and the incidence of cancer increases with age.^30^ The incidence of tumors in elderly individuals is higher than that in young individuals, and over 65‐year‐old cancer patients are often treated with ICIs.^31^ In addition, it may also be related to the combination of other chronic diseases in elderly individuals, which destroy and affect the body's immune system, resulting in low immune function.^32^ Elderly patients are at greater risk for cardiotoxicity due to a pre‐existing phenotypic susceptibility characterized by chronic heart disease.^33^

This study showed that treatment with ICIs was significantly associated with the frequency of reported adverse arrhythmic events (ROR 1.12, 95% CI 1.08–1.17), which is consistent with previous studies on supraventricular arrhythmia.^19^ It is over‐reported that anti‐PD‐1/anti‐PD‐L1 drugs induce arrhythmias, but the signal intensity is weak. ICI‐related arrhythmic events were over‐reported with anti‐PD‐1/PD‐L1 versus anti‐CTLA‐4 monotherapy (1.06 [0.91–1.24] and 1.28 [1.07–1.53], respectively). Furthermore, there was no increased risk with combination therapy compared with monotherapy (ROR: 0.93 [0.83–1.05]). In our analysis, of all monotherapies, anti‐PD‐L1 had the strongest association with ICI‐related arrhythmic events, which is consistent with findings from previous studies.^23^ A risk for arrhythmic events was not associated with anti‐CTLA‐4 drugs (ROR: 1.04 [0.90–1.21]). This is probably because anti‐CTLA‐4 drugs have more adverse effects than anti‐PD‐1/anti‐PD‐L1 drugs; these adverse effects mainly involve the skin, gastrointestinal tract, and endocrine system.^34^, ^35^ Furthermore, anti‐PD‐1/anti‐PD‐L1 drugs had higher susceptibility to cardiotoxicity than anti‐CTLA‐4,^23^ which is similar to our findings. Due to the lack of researches on immunotherapy‐induced arrhythmia, it is necessary to explore and clarify the relationship between anti‐CTLA‐4 drugs and arrhythmia. Furthermore, surprisingly, combination therapy was also not associated with arrhythmia risk. Previous studies have shown an increased risk of myocarditis with ICI combination therapy. However, Sznol et al.^36^ did not find myocarditis in the irAEs of 448 patients treated with ipilimumab in combination with nivolumab. There is some controversy over whether ICI combination therapy increases cardiovascular toxicity. We speculate that this may be related to the low reported rate of arrhythmic events induced by the combination therapy and the increased number of irAEs induced by the combination therapy of anti‐PD‐1 and anti‐CTLA‐4.^37^, ^38^ The reasons for the weak and absent signal of anti‐PD‐1 in combination with anti‐CTLA‐4 need to be further explored. It is worth noting that arrhythmia is a common comorbidity in cancer patients, a common complication of anticancer therapy and may occur simultaneously with other irAEs. Therefore, we need to pay attention and differentiate the cases where the arrhythmia is secondary to other concurrent irAEs or caused by the ICI treatment itself.^19^ The diagnosis of ICI‐related arrhythmias remains a diagnosis of exclusion, requiring the exclusion of other known causes. In general, the gold standard for a definitive diagnosis of ICI associated myocarditis is histopathological evidence of inflammation with biopsy. However, due to the difficulty in obtaining target tissue and safety considerations, biopsies may not be available for all patients. Although there are currently guidelines for diagnosing and assessing the severity of ICI associated myocarditis, ICI‐related arrhythmias do not have clear diagnostic criteria, and in such cases, only clinicians are relied on to describe and judge the ADE. In addition, early identification and timely management of patients presenting with ICI‐associated myocarditis is necessary. To date, few validated methods or biomarkers have been proposed for the prediction of patients with ICI‐associated myocarditis. Recently, Xu et al.^39^ found that significant electrocardiogram and echocardiographic abnormalities in ICI‐associated myocarditis can be used as a cue for major adverse cardiac events and have clinical predictive value.

Our further study on adverse arrhythmic events found that sudden death and complete AV block were ADEs that were significantly associated with ICI‐related arrhythmic events and had stronger signal intensity. There have been reports of patients treated with ICIs experiencing life‐threatening AV block or ventricular tachyarrhythmias. A previous study^40^ found that conduction disturbances were associated with increased cardiovascular mortality in patients treated with ICIs. A 2018 systematic review found that 10% of cardiotoxic events associated with ICIs were AV block or conduction disease, which caused death in 50% of patients.^38^ In addition, ICI‐induced arrhythmias may coexist with myocarditis or AV block, manifesting as varying degrees of heart block, bradycardia, or sudden cardiac death due to total heart block.^40^ Therefore, it is clinically necessary to maintain vigilance against complete AV block and strengthen supervision, and further research is urgently needed.

Immune checkpoint inhibitor‐related arrhythmic events in our analysis occurred early after the onset of ICIs. The median time from initiation of ICI therapy to the onset of an adverse arrhythmic event was 32 days (11–84), earlier than the median onset time of atrial fibrillation reported by Jain et al. (3.37 months; IQR 1.4–7.65 months), and the median onset time of conduction disturbances (4.83 months; IQR 1.62–11.36 months).^41^ In addition, we found that the TTO of fatal cases (30 days [IQR] 11–73.75) was significantly earlier than that of non‐fatal cases (33 days [IQR 10.5–88.5]), with a significant difference. Cardiovascular toxicity is most often detected in a month or so, demonstrating the importance of monitoring arrhythmias and intervening them during this high‐risk window.

Furthermore, we evaluated the risk of death due to adverse arrhythmic events induced by ICI. In both fatal and non‐fatal cases, the proportion of fatal arrhythmias and non‐fatal arrhythmias was similar between ICI monotherapy and ICI combined therapy. A fatal arrhythmia was more likely to occur in patients over 65 years of age. Shah et al.^42^ found that older patients with irAEs were associated with more deaths and longer hospital stays, which is consistent with our results. We speculate that older patients may be more prone to complications from cardiovascular toxicity or high doses of steroids, leading to higher mortality. In addition, there were significant differences in mortality among different reporting regions, with the highest being reported in Asia (39.31%, 160/407). Peng et al.^43^ believed that the incidence of irAEs, especially pulmonary toxicity and hepatotoxicity, was relatively high in Asian populations. The association of incidence or severity between Asian and Caucasian patients is unclear.^44^ The differences in mortality between reported regions did not suggest a causal relationship between race and death. Due to ICIs' high mortality rate, Asian populations should pay special attention to the risk of arrhythmia induced by them. There is still more data needed to identify patient‐specific risk factors.

We also investigated the prognosis of ICI‐associated adverse arrhythmic events. The results showed that the associated death rate was 26.07%. To our knowledge, this is the first time that the proportion of arrhythmic death outcomes has been described. A study conducted by Rubio et al.^45^ to analyze the incidence and characteristics of cardiac irAEs found that 1.3% of patients developed cardiac irAEs, among which myocarditis was the most common (50.8%), and 15 patients died of cardiac irAEs (24.6%). Previous studies^18^ found that cardiovascular irAEs were severe in most cases (>80%), with death occurring in 61 of 122 cases of myocarditis (50%), 20 of 95 cases of pericardial disease (21%) and 5 of 82 cases of vasculitis (6%). Interestingly, patients with arrhythmias induced by ipilimumab plus nivolumab combination therapy had lower mortality than patients with arrhythmias induced by ipilimumab monotherapy. In previous studies, irAEs occurred with a higher incidence and severity in combination therapy, contrary to this result.^30^, ^46^ There may be several reasons for this discrepancy. On the one hand, it is important to note that real‐world data differs from clinical trial data, which require strict patient selection criteria based on sex, age, and health status. Immune combination therapy is more commonly administered to relatively young patients to overcome potential adverse effects. On the other hand, the dose of combination therapy is usually reduced compared to that of ICI monotherapy.^47^, ^48^

Several limitations are present in this study. First, the FAERS database is the ADE self‐reporting system established by the FDA of the United States, and it has many problems, such as jumbled sources, repeated or missing information, and difficult to control confounding factors. This study only showed a statistical correlation between ICIs and arrhythmia but could not prove a causal link, which needs to be explored in further clinical trials. Second, the reporting of ADEs in the FAERS database can be subjective, and its identification and reporting are not strictly regulated. In addition, there is a lack of information on the number of people using drugs, so the incidence of ADEs cannot be calculated, and only reflects the intensity signals. The absence of total exposure data makes it impossible to calculate mortality rates, considering that deaths may also be caused by underlying diseases, jointly reported irAEs and other events. Last, the ADE reports in the FAERS database are mainly from European and American countries, and there is a certain ethnic deviation.

Although ROR, PRR and IC methods have high sensitivity and simple calculation methods, they are prone to false positives under specific conditions. The ADE signals obtained by disproportionality method calculation indicate that the ICIs is statistically associated with the arrhythmic events, rather than biologically associated, and do not prove that the ICIs has an inevitable causal relationship with the arrhythmic events, and its association needs to be confirmed by further clinical study evaluation.

## 5 CONCLUSIONS

This study describes the clinical features of arrhythmic events in patients treated with ICIs and conducts a disproportionality analysis based on FAERS database, suggesting that ICIs may increase the risk of arrhythmias and lead to severe outcomes, and that they tend to occur soon after the start of ICI treatment. Further research is needed to elucidate the mechanisms of arrhythmias associated with ICIs, to assess case causality and to identify and confirm these risks more comprehensively and broadly.

## AUTHOR CONTRIBUTIONS

**Yunwei Liu:** Conceptualization (equal); formal analysis (equal); methodology (equal); writing – original draft (equal). **Yanxin Chen:** Data curation (equal); formal analysis (equal); software (equal). **Zhimin Zeng:** Project administration (equal); supervision (equal). **Anwen Liu:** Project administration (lead); supervision (equal); writing – review and editing (lead).

## FUNDING INFORMATION

This work was supported by the National Nature Science Foundation (grant number 82060577).

## CONFLICT OF INTEREST

This research was conducted without the involvement of any commercial or financial interests.

## ETHICS APPROVAL AND CONSENT TO PARTICIPATE

Not applicable.

## Supporting information


Appendix S1.

## Data Availability

Data are presented in tables and figures, and other related information can be requested from the corresponding author.

## References

[1] Anderson NM , Simon MC . The tumor microenvironment. Curr Biol. 2020; 30(16): R921‐R925. 32810447 10.1016/j.cub.2020.06.081 PMC8194051

[2] Remon J , Passiglia F , Ahn MJ , et al. Immune checkpoint inhibitors in thoracic malignancies: review of the existing evidence by an IASLC expert panel and recommendations. J Thorac Oncol. 2020; 15(6): 914‐947. 32179179 10.1016/j.jtho.2020.03.006

[3] Ribas A , Wolchok JD . Cancer immunotherapy using checkpoint blockade. Science. 2018; 359(6382): 1350‐1355. 29567705 10.1126/science.aar4060 PMC7391259

[4] Marin‐Acevedo JA , Kimbrough EO , Lou Y . Next generation of immune checkpoint inhibitors and beyond. J Hematol Oncol. 2021; 14(1): 45. 33741032 10.1186/s13045-021-01056-8 PMC7977302

[5] Vaddepally RK , Kharel P , Pandey R , Garje R , Chandra AB . Review of indications of FDA‐approved immune checkpoint inhibitors per NCCN guidelines with the level of evidence. Cancers (Basel). 2020; 12(3): 738. 32245016 10.3390/cancers12030738 PMC7140028

[6] Sharma P , Siddiqui BA , Anandhan S , et al. The next decade of immune checkpoint therapy. Cancer Discov. 2021; 11(4): 838‐857. 33811120 10.1158/2159-8290.CD-20-1680

[7] Sullivan RJ , Weber JS . Immune‐related toxicities of checkpoint inhibitors: mechanisms and mitigation strategies. Nat Rev Drug Discov. 2022; 21(7): 495‐508. 34316029 10.1038/s41573-021-00259-5

[8] Lyon AR , Yousaf N , Battisti NML , Moslehi J , Larkin J . Immune checkpoint inhibitors and cardiovascular toxicity. Lancet Oncol. 2018; 19(9): e447‐e458. 30191849 10.1016/S1470-2045(18)30457-1

[9] Mahmood SS , Fradley MG , Cohen JV , et al. Myocarditis in patients treated with immune checkpoint inhibitors. J Am Coll Cardiol. 2018; 71(16): 1755‐1764. 29567210 10.1016/j.jacc.2018.02.037 PMC6196725

[10] Moslehi JJ , Salem JE , Sosman JA , Lebrun‐Vignes B , Johnson DB . Increased reporting of fatal immune checkpoint inhibitor‐associated myocarditis. Lancet. 2018; 391(10124): 933. 10.1016/S0140-6736(18)30533-6 PMC6668330 29536852

[11] Escudier M , Cautela J , Malissen N , et al. Clinical features, management, and outcomes of immune checkpoint inhibitor‐related cardiotoxicity. Circulation. 2017; 136(21): 2085‐2087. 29158217 10.1161/CIRCULATIONAHA.117.030571

[12] Kichloo A , Aljadah M , Vipparla N , et al. Pembrolizumab‐induced myocarditis leading to persistent atrial arrhythmias and a Cascade of complications: a therapeutic dilemma. Am J Ther. 2020; 28(5): e606‐e608. 33136576 10.1097/MJT.0000000000001287

[13] Reddy N , Moudgil R , Lopez‐Mattei JC , et al. Progressive and reversible conduction disease with checkpoint inhibitors. Can J Cardiol. 2017; 33(10): 1335.e13‐1335.e15. 10.1016/j.cjca.2017.05.026 28822650

[14] Katsume Y , Isawa T , Toi Y , et al. Complete atrioventricular block associated with pembrolizumab‐induced acute myocarditis: the need for close cardiac monitoring. Intern Med. 2018; 57(21): 3157‐3162. 29877257 10.2169/internalmedicine.0255-17 PMC6262691

[15] Waliany S , Zhu H , Wakelee H , et al. Pharmacovigilance analysis of cardiac toxicities associated with targeted therapies for metastatic NSCLC. J Thorac Oncol. 2021; 16(12): 2029‐2039. 34418561 10.1016/j.jtho.2021.07.030

[16] Salem JE , Nguyen LS , Moslehi JJ , et al. Anticancer drug‐induced life‐threatening ventricular arrhythmias: a World Health Organization pharmacovigilance study. Eur Heart J. 2021; 42(38): 3915‐3928. 34370839 10.1093/eurheartj/ehab362 PMC8677441

[17] Böhm R , von Hehn L , Herdegen T , et al. OpenVigil FDA‐inspection of U.S. American adverse drug events pharmacovigilance data and novel clinical applications. PLoS One. 2016; 11(6): e0157753. 27326858 10.1371/journal.pone.0157753 PMC4915658

[18] Lee A , Duggan S , Deeks ED . Cemiplimab: a review in advanced cutaneous squamous cell carcinoma [published correction appears in drugs. 2020 Jun;80(9):939]. Drugs. 2020; 80(8): 813‐819. 32306208 10.1007/s40265-020-01302-2

[19] Salem JE , Manouchehri A , Moey M , et al. Cardiovascular toxicities associated with immune checkpoint inhibitors: an observational, retrospective, pharmacovigilance study. Lancet Oncol. 2018; 19(12): 1579‐1589. 30442497 10.1016/S1470-2045(18)30608-9 PMC6287923

[20] Fan Q , Hu Y , Yang C , Zhao B . Myocarditis following the use of different immune checkpoint inhibitor regimens: a real‐world analysis of post‐marketing surveillance data. Int Immunopharmacol. 2019; 76: 105866. 31491729 10.1016/j.intimp.2019.105866

[21] Mascolo A , Scavone C , Ferrajolo C , et al. Immune checkpoint inhibitors and cardiotoxicity: an analysis of spontaneous reports in eudravigilance. Drug Saf. 2021; 44(9): 957‐971. 34145536 10.1007/s40264-021-01086-8 PMC8370948

[22] Ma Z , Pei J , Sun X , et al. Pericardial toxicities associated with immune checkpoint inhibitors: a pharmacovigilance analysis of the FDA adverse event reporting system (FAERS) database. Front Pharmacol. 2021; 12: 663088. 34276364 10.3389/fphar.2021.663088 PMC8283181

[23] Chen C , Chen T , Liang J , et al. Cardiotoxicity induced by immune checkpoint inhibitors: a pharmacovigilance study from 2014 to 2019 based on FAERS. Front Pharmacol. 2021; 12: 616505. 33643048 10.3389/fphar.2021.616505 PMC7907652

[24] Ma R , Wang Q , Meng D , Li K , Zhang Y . Immune checkpoint inhibitors‐related myocarditis in patients with cancer: an analysis of international spontaneous reporting systems. BMC Cancer. 2021; 21(1): 38. 33413213 10.1186/s12885-020-07741-0 PMC7791701

[25] Conforti F , Pala L , Bagnardi V , et al. Cancer immunotherapy efficacy and patients' sex: a systematic review and meta‐analysis. Lancet Oncol. 2018; 19(6): 737‐746. 29778737 10.1016/S1470-2045(18)30261-4

[26] Hu JR , Florido R , Lipson EJ , et al. Cardiovascular toxicities associated with immune checkpoint inhibitors. Cardiovasc Res. 2019; 115(5): 854‐868. 30715219 10.1093/cvr/cvz026 PMC6452314

[27] Henley SJ , Richards TB , Underwood JM , et al. Lung cancer incidence trends among men and women‐United States, 2005–2009. MMWR Morb Mortal Wkly Rep. 2014; 63(1): 1‐5. 24402465 PMC5779336

[28] Sung H , Ferlay J , Siegel RL , et al. Global cancer statistics 2020: GLOBOCAN estimates of incidence and mortality worldwide for 36 cancers in 185 countries. CA Cancer J Clin. 2021; 71(3): 209‐249. 33538338 10.3322/caac.21660

[29] Jing Y , Zhang Y , Wang J , et al. Association between sex and immune‐related adverse events during immune checkpoint inhibitor therapy. J Natl Cancer Inst. 2021; 113(10): 1396‐1404. 33705549 10.1093/jnci/djab035

[30] Chatsirisupachai K , Lesluyes T , Paraoan L , van Loop P , de Magalhães JP . Integrative analysis of the age‐associated multi‐omic landscape across cancers. Nat Commun. 2021; 12(1): 2345. 33879792 10.1038/s41467-021-22560-y PMC8058097

[31] Loh KP , Wong ML , Maggiore R . From clinical trials to real‐world practice: immune checkpoint inhibitors in older adults. J Geriatr Oncol. 2019; 10(3): 384‐388. 30745116 10.1016/j.jgo.2019.01.023

[32] Granier C , Gey A , Roncelin S , Weiss L , Paillaud E , Tartour E . Immunotherapy in older patients with cancer. Biomed J. 2021; 44(3): 260‐271. 33041248 10.1016/j.bj.2020.07.009 PMC8358190

[33] Romitan DM , van Loop P , Rădulescu D , Berindan‐Neagoe I , et al. Cardiomyopathies and arrhythmias induced by cancer therapies. Biomedicine. 2020; 8(11): 496. 10.3390/biomedicines8110496 PMC7696637 33198152

[34] Boutros C , Tarhini A , Routier E , et al. Safety profiles of anti‐CTLA‐4 and anti‐PD‐1 antibodies alone and in combination. Nat Rev Clin Oncol. 2016; 13(8): 473‐486. 27141885 10.1038/nrclinonc.2016.58

[35] El Osta B , Hu F , Sadek R , et al. Not all immune‐checkpoint inhibitors are created equal: meta‐analysis and systematic review of immune‐related adverse events in cancer trials. Crit Rev Oncol Hematol. 2017; 119: 1‐12. 29065979 10.1016/j.critrevonc.2017.09.002

[36] Sznol M , Ferrucci PF , Hogg D , et al. Pooled analysis safety profile of nivolumab and ipilimumab combination therapy in patients with advanced melanoma. J Clin Oncol. 2017; 35(34): 3815‐3822. 28915085 10.1200/JCO.2016.72.1167

[37] Wolchok JD , Chiarion‐Sileni V , Gonzalez R , et al. Overall survival with combined nivolumab and ipilimumab in advanced melanoma. N Engl J Med. 2017; 377(14): 1345‐1356. 28889792 10.1056/NEJMoa1709684 PMC5706778

[38] Shoushtari AN , Friedman CF , Navid‐Azarbaijani P , et al. Measuring toxic effects and time to treatment failure for nivolumab plus ipilimumab in melanoma. JAMA Oncol. 2018; 4(1): 98‐101. 28817755 10.1001/jamaoncol.2017.2391 PMC5833656

[39] Xu Y , Song Y , Liu X , et al. Prediction of major adverse cardiac events is the first critical task in the management of immune checkpoint inhibitor‐associated myocarditis. Cancer Commun (Lond). 2022; 42(9): 902‐905. 35678260 10.1002/cac2.12320 PMC9456696

[40] Safi M , Ahmed H , Al‐Azab M , et al. PD‐1/PDL‐1 inhibitors and cardiotoxicity; molecular, etiological and management outlines. J Adv Res. 2020; 29: 45‐54. 33842004 10.1016/j.jare.2020.09.006 PMC8020146

[41] Jain P , Gutierrez Bugarin J , Guha A , et al. Cardiovascular adverse events are associated with usage of immune checkpoint inhibitors in real‐world clinical data across the United States. ESMO Open. 2021; 6(5): 100252. 34461483 10.1016/j.esmoop.2021.100252 PMC8403739

[42] Shah KP , Song H , Ye F , et al. Demographic factors associated with toxicity in patients treated with anti‐programmed cell death‐1 therapy. Cancer Immunol Res. 2020; 8(7): 851‐855. 32350001 10.1158/2326-6066.CIR-19-0986 PMC7334081

[43] Peng L , Wu YL . Immunotherapy in the Asiatic population: any differences from Caucasian population? J Thorac Dis. 2018; 10(Suppl 13): S1482‐S1493. 29951300 10.21037/jtd.2018.05.106 PMC5994501

[44] Lee J , Sun JM , Lee SH , Ahn JS , Park K , Ahn MJ . Are there any ethnic differences in the efficacy and safety of immune checkpoint inhibitors for treatment of lung cancer? J Thorac Dis. 2020; 12(7): 3796‐3803. 32802459 10.21037/jtd.2019.08.29 PMC7399433

[45] Rubio‐Infante N , Ramírez‐Flores YA , Castillo EC , Lozano O , García‐Rivas G , Torre‐Amione G . Cardiotoxicity associated with immune checkpoint inhibitor therapy: a meta‐analysis. Eur J Heart Fail. 2021; 23(10): 1739‐1747. 34196077 10.1002/ejhf.2289

[46] Almutairi AR , McBride A , Slack M , Erstad BL , Abraham I . Potential immune‐related adverse events associated with monotherapy and combination therapy of Ipilimumab, nivolumab, and pembrolizumab for advanced melanoma: a systematic review and meta‐analysis. Front Oncol. 2020; 10: 91. 32117745 10.3389/fonc.2020.00091 PMC7033582

[47] Hassel JC , Heinzerling L , Aberle J , et al. Combined immune checkpoint blockade (anti‐PD‐1/anti‐CTLA‐4): evaluation and management of adverse drug reactions. Cancer Treat Rev. 2017; 57: 36‐49. 28550712 10.1016/j.ctrv.2017.05.003

[48] Antonia SJ , López‐Martin JA , Bendell J , et al. Nivolumab alone and nivolumab plus ipilimumab in recurrent small‐cell lung cancer (CheckMate 032): a multicentre, open‐label, phase 1/2 trial. Lancet Oncol. 2016; 17(7): 883‐895. 27269741 10.1016/S1470-2045(16)30098-5

