# Supplementary material for: Arrhythmic events associated with immune checkpoint inhibitors therapy: A real‐world study based on the Food and Drug Administration Adverse Event Reporting System database
Source: Cancer Med. 2022 Nov 24;12(6):6637–48. doi: 10.1002/cam4.5438 (PMC10067122; doi:10.1002/cam4.5438)
Supplement: Supplementary file 1 — Appendix S1. [file CAM4-12-6637-s001.pdf]

Supplementary Table 1. Summary of FDA-approved ICIs

Target	Generic name	Brand name	Approval year
PD-1	nivolumab	OPDIVO, MDX-1106, ONO4538, BMS-936558	2014
	pembrolizumab	KEYTRUDA, MK-3475	2014
	cemiplimab	LIBTAYO, SAR439684, REGN2810	2018
PD-L1	atezolizumab	TECENTRIQ, MPDL3280A	2016
	durvalumab	IMFINZI, MEDI4736	2017
	avelumab	BAVENCIO, MSB0010718C	2017
CTLA-4	ipilimumab	YERVOY, BMS-734016, MDX-010	2011
	tremelimumab	-	-

FDA, Food and Drug Administration; ICIs, immune checkpoint inhibitors;PD-1, programmed cell death protein 1; PD-L1, programmed cell death ligand 1, CTLA-4, cytotoxic T lymphocyte-associated antigen 4.

For Review Only

Supplementary Table 2. Fourfold table of measures of disproportionality

Drug	Adverse event of interest	All other adverse events	Total
Drug of interest	a	b	a+b
All other drugs	c	d	c+d
Total	a+c	b+d	N=a+b+c+d

Supplementary Table 3. Summary of major algorithms used for signal detection.

Algorithms	Equation	Criteria
ROR	ROR=ad/c/b<br>95%CI=$e^{\ln(ROR)\pm1.96(1/a+1/b+1/c+1/d)^{\wedge}0.5}$	lower limit of 95% CI>1, N≥2
PRR	$PRR=\frac{a/(a+b)}{c/(c+d)}$<br>$SE(\ln PRR)=\sqrt{(\frac{1}{a}-\frac{1}{a+b}+\frac{1}{c}-\frac{1}{c+d})}$<br>$95\%Cl=e^{\ln(PRR)\pm1.96\sqrt{(\frac{1}{a}-\frac{1}{a+b}+\frac{1}{c}-\frac{1}{c+d})}}$	N≥3, PRR >1 **[1-2]**<br>$\chi^2\geq4$

BCPNN	χ^2=[(ad-bc)^2](a+b+c+d)/[(a+b)(c+d)(a+c)(b+d)]<br>IC=$\log_2$a(a+b+c+d)(a+c)(a+b)<br>95%CI=$e^{\ln(IC)\pm1.96(1/a+1/b+1/c+1/d)^{\wedge}0.5}$	IC025>0

ROR, reporting odds ratio; CI, confidence interval; N, the number of co-occurrences; PRR, proportional reporting ratio, χ^2 , chi-square; BCPNN, Bayesian confidence propagation neural network; IC, information component; IC025, the lower limit of the 95% two-sided CI of the IC.

Supplementary Table 4. Comparison of clinical characteristics between patients with lung cancer and patients with other indications in immune checkpoint inhibitor-related arrhythmia events

Characteristics		Lung cancer cases, n (%)	Other cancer cases, n (%)	p-value
Total		624	1321	
Gender	-			NS
	Famale	172 (27.56)	389 (29.45)	
	Male	413 (66.19)	847 (64.12)	
	Unkown	39 (6.25)	85 (6.43)	
Age	-			<0.0001
	Median	69 (63-75)	68 (59-75)	
	<18	0	7 (0.53)	
	18-64	160 (25.64)	394 (29.83)	
	≥65	357 (57.21)	693 (52.46)	
	Unkown	107 (17.15)	227 (17.18)	
Reporting year	-			<0.0001
	2011	0	19 (1.44)	
	2012	1 (0.16)	29 (2.20)	
	2013	0	24 (1.82)	
	2014	1 (0.16)	35 (2.65)	
	2015	20 (3.21)	61 (4.62)	
	2016	50 (8.01)	79 (5.98)	
	2017	85 (13.62)	149 (11.28)	
	2018	145 (23.24)	169 (12.79)	
	2019	117 (18.75)	228 (17.26)	
	2020	101 (16.19)	222 (16.81)	
	2021	104 (16.67)	306 (23.16)	
Reporting Region	-			<0.0001
	Europe	229 (36.70)	415 (31.42)	
	America	200 (32.05)	634 (47.99)	
	Asia	186 (29.81)	221 (16.73)	
	Oceania	8 (1.28)	45 (3.41)	
	Africa	1 (0.16)	3 (0.23)	
	Unkown	0	3 (0.23)	
Indications(TOP ten)	-			<0.0001
	Lung cancer	624	0	
	Melanoma	0	398 (30.13)	
	Tumors of urinary system	0	316 (23.92)	
	Head and neck cancer	0	71 (5.37)	
	Hematological cancer and lymphoma	0	56 (4.24)	
	Tumors of female reproductive organs	0	47 (3.56)	
	Gastrointestinal cancer	0	44 (3.33)	
	Breast cancer	0	32 (2.42)	
	Mesothelioma	0	28 (2.12)	
	Hepatocellular carcinoma	0	22 (1.67)	
Outcome	-			NS
	Death	174 (27.88)	333 (25.21)	
	Life-threatening	48 (7.69)	102 (7.72)	
	Disability	6 (0.96)	19 (1.44)	
	Hospitalization	285 (45.67)	649 (49.13)	
	Other outcomes	106 (16.99)	193 (14.61)	
	RI	1 (0.16)	5 (0.38)	
	CA	0	1 (0.08)	
	Non-Serious	4 (0.64)	19 (1.44)	
Reporter TTO	-			0.014
	Median	22 (6.5-70.5)	37 (14-85)	
	0-30	208 (33.33)	350 (26.50)	
	31-60	55 (8.81)	144 (10.90)	

For Review Only

	61-90	28 (4.49)	97 (7.34)	
	91-180	37 (5.93)	87 (6.59)	
	>180	38 (6.09)	97 (7.34)	
	Unkown	258 (41.35)	546 (41.33)	
ICI drug as suspected drug	-			<0.0001
	Monotherapy	552 (88.46)	1079 (81.68)	
	Anti-PD-1 monotherapy	421 (67.47)	723 (54.73)	
	pembrolizumab	162 (25.96)	213 (16.12)	
	Nivolumab	259 (41.51)	502 (38.00)	
	Cemiplimab	0	8 (0.61)	
	Anti-PD-L1monotherapy	120 (19.23)	189 (14.31)	
	Atezolizumab	81 (12.98)	143 (10.83)	
	Avelumab	1 (0.16)	28 (2.12)	
	Durvalumab	38 (6.09)	18 (1.36)	
	Anti-CTLA-4monotherapy	11 (1.76)	167 (12.64)	
	Ipilimumab	11 (1.76)	167 (12.64)	
	Tremelimumab	0	0	
	Combination therapy	72 (11.54)	242 (18.32)	
	Ipilimumab+nivolumab	62 (9.94)	226 (17.11)	
	Ipilimumab+pembrolizumab	0	5 (0.38)	
	Tremelimumab+Durvalumab	5 (0.80)	4 (0.30)	
	Pembrolizumab+Atezolizumab	5 (0.80)	7 (0.53)	

Supplementary Table 5. Arrhythmic events reported with ICIs from FAERS

Arrhythmic event	N	%	ROR(ROR_{025})
Sudden death	151	0.16%	4.34 (3.69)
Atrioventricular block complete	96	0.10%	4.27 (3.48)
Atrioventricular block	62	0.07%	2.56 (1.99)
Supraventricular tachycardia	76	0.08%	2.22 (1.77)
Atrial flutter	65	0.07%	2.13 (1.67)
Atrial fibrillation	576	0.62%	1.61 (1.49)
Sinus tachycardia	86	0.09%	1.77 (1.43)
Ventricular tachycardia	141	0.15%	1.61 (1.37)
Bundle branch block	5	0.01%	2.62 (1.08)
Arrhythmia supraventricular	8	0.01%	2.10 (1.05)
Ventricular arrhythmia	19	0.02%	1.39 (0.89)
Cardiac arrest	284	0.31%	0.94 (0.84)
Arrhythmia	156	0.17%	0.90 (0.77)
Conduction disorder	9	0.01%	1.43 (0.74)
Tachyarrhythmia	11	0.01%	1.26 (0.70)
Ventricular fibrillation	31	0.03%	0.83 (0.58)
Tachycardia	175	0.19%	0.64 (0.55)
Sudden cardiac death	12	0.01%	0.85 (0.48)
Sinus bradycardia	25	0.03%	0.71 (0.48)
Bundle branch block right	13	0.01%	0.79 (0.46)
Atrioventricular block second degree	9	0.01%	0.79 (0.42)
Bradycardia	89	0.10%	0.46 (0.38)
Ventricular extrasystoles	14	0.02%	0.45 (0.27)
Bundle branch block left	6	0.01%	0.39 (0.17)
Electrocardiogram qt prolonged	24	0.03%	0.18 (0.12)

For Review Only

Supplementary Table 6. Comparison of clinical characteristics of cardiac arrest with other reports in immune checkpoint inhibitor-related arrhythmia events

Characteristics		Cardiac arrest reports,n(%)	Others reports ,n(%)	p-value
Total		447	1498	
Gender	-			NS
	Famale	126 (28.19)	435 (29.04)	
	Male	291 (65.10)	969 (64.69)	
	Unkown	30 (6.71)	94 (6.28)	
Age	-			<0.0001
	Median	68 (58-75)	68 (61-75)	
	<18	2 (0.45)	5 (0.33)	
	18-64	142 (31.77)	412 (27.50)	
	≥65	209 (46.76)	841 (56.14)	
	Unkown	94 (21.03)	240 (16.02)	
Reporting year	-			<0.0001
	2011	4 (0.89)	15 (1.00)	
	2012	6 (1.34)	24 (1.60)	
	2013	4 (0.89)	20 (1.34)	
	2014	9 (2.01)	27 (1.80)	
	2015	22 (4.92)	59 (3.94)	
	2016	33 (7.38)	96 (6.41)	
	2017	55 (12.30)	179 (11.95)	
	2018	73 (16.33)	241 (16.09)	
	2019	79 (17.67)	266 (17.76)	
	2020	69 (15.44)	254 (16.96)	
	2021	93 (20.81)	317 (21.16)	
Reporting Region	-			<0.0001
	Europe	139 (31.10)	505 (33.71)	
	America	175 (39.15)	659 (43.99)	
	Asia	126 (28.19)	281 (18.76)	
	Oceania	5 (1.12)	48 (3.20)	
	Africa	2 (0.45)	2 (0.13)	
	Unkown	0	3 (0.20)	
Indications(TOP ten)	-			<0.0001
	Lung cancer	138 (30.87)	486 (32.44)	
	Melanoma	82 (18.34)	316 (21.09)	
	Tumors of urinary system	79 (17.67)	237 (15.82)	
	Head and neck cancer	35 (7.83)	36 (2.40)	
	Hematological cancer and lymphoma	15 (3.36)	41 (2.74)	
	Tumors of female reproductive organs	11 (2.46)	36 (2.40)	
	Gastrointestinal cancer	16 (3.58)	28 (1.87)	
	Breast cancer	5 (1.12)	27 (1.80)	
	Mesothelioma	3 (0.67)	25 (1.67)	
	Hepatocellular carcinoma	6 (1.34)	16 (1.07)	
Outcome	-			<0.0001
	Death	299 (66.89)	208 (13.89)	
	Life-threatening	42 (9.40)	108 (7.21)	
	Disability	3 (0.67)	22 (1.47)	
	Hospitalization	94 (21.03)	840 (56.07)	
	Other outcomes	7 (1.57)	292 (19.49)	
	RI	2 (0.45)	4 (0.27)	
	CA	0	1 (0.07)	
	Non-Serious	0	23 (1.54)	
Reporter TTO	-			<0.0001
	Median	39 (14-90.5)	31 (10-81.25)	
	0-30	121 (27.07)	437 (29.17)	
	31-60	41 (9.17)	158 (10.55)	
	61-90	37 (8.28)	88 (5.87)	
	91-180	31 (6.94)	93 (6.21)	
	>180	35 (7.83)	100 (6.68)	
	Unkown	182 (40.72)	622 (41.52)	
ICI drug as suspected drug	-			NS
	Monotherapy	361 (80.76)	1270 (84.78)	
	Anti-PD-1 monotherapy	249 (55.70)	895 (59.75)	
	pembrolizumab	80 (17.90)	295 (19.69)	
	Nivolumab	167 (37.36)	594 (39.65)	
	Cemiplimab	2 (0.45)	6 (0.40)	
	Anti-PD-L1monotherapy	68 (15.21)	241 (16.09)	
	Atezolizumab	45 (10.07)	179 (11.95)	
	Avelumab	8 (1.79)	21 (1.40)	
	Durvalumab	15 (3.36)	41 (2.74)	
	Anti-CTLA-4monotherapy	44 (9.84)	134 (8.95)	
	Ipilimumab	44 (9.84)	134 (8.95)	
	Tremelimumab	0	0	
	Combination therapy	86 (19.24)	228 (15.22)	

For Review Only

	Ipilimumab+nivolumab	80 (17.90)	208 (13.89)
	Ipilimumab+pembrolizumab	0	5 (0.33)
	Tremelimumab+Durvalumab	3 (0.67)	6 (0.40)
	Pembrolizumab+Atezolizumab	3 (0.67)	9 (0.60)

References

1. Frent I, Leucuta D, Bucsa C, et al. A Description of Acute Renal Failure and Nephrolithiasis Associated With Sodium-Glucose Co-Transporter 2 Inhibitor Use: A VigiBase Study. Front Pharmacol. 2022 Aug 8;13:925805.

2. Slattery J, Alvarez Y, Hidalgo A. Choosing thresholds for statistical signal detection with the proportional reporting ratio. Drug Saf. 2013;36(8):687-692.

For Review Only
